# Lack of association of a variable number of aspartic acid residues in the asporin gene with osteoarthritis susceptibility: case-control studies in Spanish Caucasians

**DOI:** 10.1186/ar1920

**Published:** 2006-03-10

**Authors:** Julio Rodriguez-Lopez, Manuel Pombo-Suarez, Myriam Liz, Juan J Gomez-Reino, Antonio Gonzalez

**Affiliations:** 1 Laboratorio de Investigacion 2 and Rheumatology Unit, Hospital Clinico Universitario de Santiago, Santiago de Compostela, Spain; 2 Department of Medicine, University of Santiago de Compostela, Spain

## Abstract

A recent genetic association study has identified a microsatellite in the coding sequence of the asporin gene as a susceptibility factor for osteoarthritis (OA). Alleles of this microsatellite determine the variable number of aspartic acid residues in the amino-terminal end of the asporin protein. Asporin binds directly to the growth factor transforming growth factor beta and inhibits its anabolic effects in cartilage, which include stimulation of collagen and aggrecan synthesis. The OA-associated allele, with 14 aspartic acid residues, inhibits the anabolic effects of transforming growth factor beta more strongly than other asporin alleles, leading to increased OA liability. We have explored whether the association found in several cohorts of Japanese hip OA and knee OA patients was also present in Spanish Caucasians. We studied patients that had undergone total joint replacement for primary OA in the hip (*n *= 303) or the knee (*n *= 188) and patients with hand OA (*n *= 233), and we compared their results with controls (*n *= 294) lacking overt OA clinical symptoms. No significant differences were observed in any of the multiple comparisons performed, which included global tests of allele frequency distributions and specific comparisons as well as stratification by affected joint and by sex. Our results, together with reports from the United Kingdom and Greece, indicate that the stretch of aspartic acid residues in asporin is not an important factor in OA susceptibility among European Caucasians. It remains possible that lifestyle, environmental or genetic differences allow for an important effect of asporin variants in other ethnic groups as has been reported in the Japanese, but this should be supported by additional studies.

## Introduction

Current concepts in osteoarthritis (OA) imply an imbalance of cartilage anabolic and catabolic processes in response to mechanical stress with the participation of inflammatory mediators [1]. Recent investigation has also shown that genetic factors account for a substantial portion of OA etiology [2,3]. These two considerations contribute to the interest of a recent report showing that a variant of asporin (ASPN) is a susceptibility factor for OA [4]. This association points to a defect in the anabolic side of cartilage homeostasis, identifies ASPN and transforming growth factor beta as important molecular players in this process, and suggests that regulatory genetic variants are important in OA susceptibility [5,6].

ASPN is a new member of the small leucin-rich proteoglycan subfamily of proteins [7,8] that is expressed at low levels in normal cartilage but that is notably increased in OA cartilage [4]. Variant ASPN proteins are due to a microsatellite in the ASPN coding sequence that determines a variable number of aspartic acid (D) residues in the amino terminal region. The D14 allele is associated with increased OA susceptibility in the Japanese (odds ratio = 1.66–2.49, depending on the cohort) [4] due to its strong inhibition of the cartilage anabolic effects induced by transforming growth factor beta [4]. Given the significance of these results, we explored the ASPN effect in Spanish patients with severe knee or hip OA or with hand OA, but we did not find association. This result is similar to findings in UK Caucasians [9] and in the Greek population [10], and together the observations indicate that the ASPN microsatellite does not have a significant effect on OA susceptibility in European Caucasians.

## Materials and methods

### Patients and controls

Patients were selected from consecutive patients undergoing total hip replacement (THR) or total knee replacement (TKR) and patients complaining of hand OA that were followed in the Rheumatology Unit. THR and TKR patients were included only if surgery has been performed at ages ranging from 55 to 75 years and if a rheumatologist considered them to suffer from severe primary OA after compatible evaluation and exclusion of the following confounding factors: inflammatory, infectious, traumatic or congenital joint pathology and lesions due to crystal deposition or osteonecrosis. Evaluation of patients included an interview with each patient specifically for this study and a review of the clinical history and a review of radiographs. Analysis of synovial fluid was not a requisite. Obesity and occupational strain were not considered exclusion causes. Patients with hand OA were required to fulfill American College of Rheumatology classification criteria [11].

The final numbers of patients in each group were 303 THR patients (183 women, 120 men), 188 TKR patients (153 women, 35 men) and 232 hand OA patients (205 women, 27 men). We recruited controls among subjects older than 55 years undergoing preoperative workup for elective surgeries other than joint surgery. We restricted analysis to the 35.5% of controls (294/828 donors, 115 women and 179 men) that did not show clinical manifestations of OA (absence of chronic pain or restriction of mobility in the two years before recruitment, no hand enlargements or deformities, and no previous medical evaluation as OA). Radiographic exploration was not performed in controls.

This study was approved by the Ethical Committee for Clinical Research of Galicia and all cases and controls gave their written informed consent to participate. All participants were of Spanish ancestry and resided in the reference area of the hospital.

### Genotyping

Peripheral blood DNA was used to genotype the D-repeat microsatellite by PCR with the primers 5'-FAM-TGGCTTTGTGCTCTGCCAAACC-3' and 5'-TCTGAGCAATGTACAACTCGTG-3'. The size of the fluorescence-labeled products was determined by capillary electrophoresis on an ABI prism 3100 Avant Genetic Analyzer (Applied Biosystems, Foster City, CA, USA). Several samples with different genotypes were sequenced to test the accuracy of genotyping with the Big Dye Ready Reaction Kit (Applied Biosystems).

### Statistical analysis

Allele frequencies and their 95% confidence intervals were calculated. Comparison of allele frequencies was carried out using the 2 × *n *contingency table after collapsing columns with low frequency, as implemented in the T2 option of the Clump software that follows a Monte Carlo approach [12]. Post-hoc power was determined with the GPower software [13] for each comparison of the D14 and D13 allelic frequencies between patients and controls.

## Results and discussion

This study explored whether the sound association between the ASPN microsatellite and OA in the Japanese population [4] was also present in the Spanish population. We therefore searched for evidence of association in multiple ways. First, the allelic frequency distribution of the microsatellite was compared between the three groups of patients separated by the affected joint and the controls (Table 1). None of these three comparisons showed differences. All *P *values were far larger than the threshold for significance: TKR patients versus controls, *P *= 0.6; THR patients versus controls, *P *= 0.5; and hand OA patients versus controls, *P *= 0.9.

Similar results were obtained after stratifying by sex (Table 1). Female patients and controls had similar allele frequencies (TKR patients versus controls, *P *= 0.9; THR patients versus controls, *P *= 0.9; and hand OA patients versus controls, *P *= 0.8), as well as male patients and controls (TKR patients versus controls, *P *= 0.6; THR patients versus controls, *P *= 0.3; hand OA patients versus controls, *P *= 0.4). We also pooled data from TKR and THR patients as both patient groups were associated with ASPN in the Japanese population, but even for this larger group there was no difference with the controls (*P *= 0.8). A similar result was observed if all the OA patients were taken together (THR + TKR + hand OA = 723 patients, *P *= 0.8).

These results are in clear contrast with the association described in several cohorts of Japanese subjects, where the frequency distributions of this microsatellite were consistently different between patients with knee OA or hip OA and controls [4], but they were similar to recent results in the UK population [9] and the Greek population [10].

The differences in the ASPN microsatellite frequency distributions reported in the Japanese study were due mainly to an increased frequency of the D14 allele and to a lesser extent due to a decreased frequency of the D13 allele in OA patients [4]. The Greek study also found a significant decrease of the D13 allele in TKR patients but there was no change in the D14 frequency. The UK study did not find significant differences but observed a tendency in the same sense as the Japanese study: an increase of the D14 allele and a decrease of the D13 allele in THR and TKR patients [9]. We therefore specifically tested these two alleles for differences in our subjects (Table 2).

No differences were detected in the D14 allele frequencies between controls and TKR patients, THR patients, both TKR and THR patients together, or hand OA patients (this later group had not been included in previous studies). We did not even observe a tendency to a higher frequency of the D14 allele in patients. The same pattern was found when this analysis was repeated after stratifying by sex. In the same way, the frequency of the D13 allele was similar in all patient groups and in controls (Table 2) and no difference was observed after stratifying by sex. Finally, as the most sensitive test, we considered only the D13 and the D14 alleles and compared their relative frequencies in each patient group with their frequencies in the controls, but again no differences were found (Table 2).

Even with this later comparison that gave the largest difference in the Japanese population, we did not find evidence of association of OA with the ASPN microsatellite in our patients. The present study has enough post-hoc power (>0.8), however, to detect a D14 effect of the size found in the Japanese [4], with *P *< 0.05. The power for the comparisons of THR patients versus controls and TKR patients versus controls is 0.83 and 0.94 for the D14 allele frequencies and is 0.90 and 0.98 for the D14 versus D13 frequencies, respectively. The comparison lacks power to detect a decrease of the D13 allele in OA patients, similar to the Japanese study (power is 0.16 for the comparison of THR patients versus controls and is 0.29 for the comparison of TKR patients versus controls), probably due to the weak effect of this allele (odds ratio = 0.84 both for hip OA and knee OA in the Japanese population).

Our results were similar to the UK study [9] except for a significant increase of the D14 allele in UK THR males that did not persist after correction for the multiple tests performed. In contrast, our patients showed a nonsignificant decrease of this allele in THR males (10.4%, 95% confidence interval 3.4–17.4% in THR males versus 13.4%, 95% confidence interval 5.6–21.2% in male controls). The D14 allele was also similar in Greek TKR patients and controls [10]. The Greek TKR patients, however, showed significantly increased frequencies of the D15 and D18 alleles, which were not found in our study (Table 1) or in the other two larger studies, and which did not remain significant after accounting for the multiple comparisons performed. The overall results therefore indicate that the ASPN microsatellite is not associated with OA susceptibility in European Caucasians. Discrepancies between the Japanese and Caucasians could be multifactorial, involving differences in disease phenotype, in lifestyle, and in environmental and genetic factors.

## Conclusion

There was no detectable effect of variation in the number of D residues in ASPN in hip OA, knee OA or hand OA susceptibility in Spanish Caucasians. This result reinforces the overall lack of association reported in other studies in Caucasians. The association between variation in the number of D residues and OA described in the Japanese study is robust, however, and is not refuted by these studies, but it will require confirmation in the same ethnic population. Therefore, although it remains possible that ASPN could be a crucial modulator in OA by fine-tuning transforming growth factor beta in the repair of damaged cartilage, this effect will be limited to some ethnic groups or environmental settings.

## Abbreviations

ASPN = asporin; D = aspartic acid; OA = osteoarthritis; PCR = polymerase chain reaction; THR = total hip replacement; TKR = total knee replacement.

## Competing interests

The authors declare that they have no competing interests.

## Authors' contributions

All authors contributed to the final manuscript. In addition, JR-L genotyped the samples and participated in the design and analysis of the study, MP-S, ML and JJG-R evaluated the patients, and AG coordinated the study and participated in its design and analysis.

## Figures and Tables

**Table 1:** Frequencies of the asporin microsatellite alleles in Spanish controls and each of the patient groups

	Allele							
Group	D12	D13	D14	D15	D16	D17	D18	D19

Controls								
All controls (*n *= 294)	31(5.3)	248 (42.2)	74 (12.6)	150 (25.5)	55 (9.3)	12 (2.0)	10 (1.7)	8 (1.3)
Males (*n *= 179)	22 (6.15)	154 (43.0)	48 (13.4)	90 (25.1)	29 (8.1)	5 (1.4)	6 (1.7)	4 (1.1)
Females (*n *= 115)	9 (3.9)	94 (40.9)	26 (11.3)	60 (26.1)	26 (11.3)	7 (3.0)	4 (1.7)	4 (1.7)
Total hip replacement								
All patients (*n *= 303)	22 (3.6)	262 (43.3)	59 (9.7)	156 (25.7)	68 (11.2)	13 (2.1)	13 (2.1)	13(2.1)
Males (*n *= 120)	7 (2.9)	105 (43.7)	25 (10.4)	64 (26.7)	20 (8.3)	6 (2.5)	9 (3.7)	4 (1.7)
Females (*n *= 183)	15 (4.1)	157 (42.9)	34 (9.3)	92 (25.1)	48 (13.1)	7 (1.9)	4 (1.1)	9 (2.5)
Total knee replacement								
All patients (*n *= 188)	14 (3.7)	156 (41.5)	56 (14.9)	93 (24.7)	30 (7.9)	14 (3.7)	7 (1.8)	6 (1.6)
Males (*n *= 35)	3 (4.3)	36 (51.4)	11 (15.7)	14 (20.0)	4 (5.7)	0 (0.0)	2 (2.8)	0 (0.0)
Females (*n *= 153)	11 (3.6)	120 (39.2)	45 (14.7)	79 (25.8)	26 (8.5)	14 (4.6)	5 (1.6)	6 (2.0)
Hand osteoarthritis								
All patients (*n *= 232)	16 (3.4)	209 (44.8)	57 (12.2)	113 (24.2)	46 (9.9)	13 (2.8)	8 (1.7)	4 (0.8)
Males (*n *= 27)	2 (3.7)	18 (33.3)	6 (11.1)	19 (35.2)	6 (11.1)	0 (0.0)	2 (3.7)	1(1.8)
Females (*n *= 205)	14 (3.4)	189 (46.1)	51 (12.4)	94 (22.9)	40 (9.8)	13 (3.2)	6 (1.5)	3 (0.7)

Values presented the number of alleles (%).

**Table 2:** Comparisons of the D14 and D13 allelic frequencies of the asporin microsatellite between Spanish osteoarthritis patients and controls represented as *P *values

	Comparison		
Group	D14 versus others	D13 versus others	D14 versus D13

Total hip replacement (*n *= 303)	0.12	0.71	0.15
Total knee replacement (*n *= 188)	0.31	0.83	0.37
Total hip replacement + total knee replacement (*n *= 597)	0.61	0.88	0.63
Hand osteoarthritis (*n *= 233)	0.86	0.38	0.65
